# Editorial: Semantic Algorithms in the Assessment of Attitudes and Personality

**DOI:** 10.3389/fpsyg.2021.720559

**Published:** 2021-07-23

**Authors:** Jan Ketil Arnulf, Kai R. Larsen, Øyvind Lund Martinsen, Kim F. Nimon

**Affiliations:** ^1^BI Norwegian Business School, Oslo, Norway; ^2^Leeds School of Business, University of Colorado, Boulder, CO, United States; ^3^Soules College of Business, The University of Texas at Tyler, Tyler, TX, United States

**Keywords:** latent semantic analysis, survey research, organizational behavior, voting behavior, trust, motivation, clinical psychology, artificial intelligence

The methodological tools available for psychological and organizational assessment are rapidly advancing through natural language processing (NLP). Computerized analyses of texts are increasingly available as extensions of traditional psychometric approaches. The present Research Topic is recognizing the contributions but also the challenges in publishing such inter-disciplinary research. We therefore sought to provide an open-access avenue for cutting-edge research to introduce and illustrate the various applications of semantics in the assessment of attitudes and personality. The result is a collection of empirical contributions spanning from assessment of psychological states through methodological biases to construct identity detection.

To understand previous research leading up to this issue, one important starting point was the application of machine learning to the assessment of attitudes measured by Larsen et al. (2008). Observing how the output from semantic algorithms could identify high correlations among items, Larsen et al. (2008, p. 3) introduced a mechanism to check for language-driven survey results:

“Manifest validity is expected to support researchers during the data analysis stage in that researchers can compare measures of manifest validity (evaluating the extent of semantic difference between different scales) to item correlations computed from actual responses. In cases where there is little difference between distances proposed by correlation coefficients, the respondents are more likely to have employed shallow processing during questionnaire analysis.”

Since then, researchers have expanded the use of semantic similarity of scale items to explore survey responses in a number of ways. Studies have shown that semantics may predict survey responses in organizational behavior (Arnulf et al., 2014, 2018c), leadership (Arnulf and Larsen, 2015, Arnulf et al., 2018b,d), employee engagement (Nimon et al., 2016), technology acceptance (Gefen and Larsen, 2017), and intrinsic motivation (Arnulf et al., 2018a).

In a parallel line of previous research, semantic analysis has been used to complement and extend data from traditional rating scales (e.g., Nicodemus et al., 2014; Bååth et al., 2019; Garcia et al., 2020, Kjell et al., 2019). Since semantic analysis can detect overlap among items and rating scales, they can be used to map relationships and overlap between existing or new scales (e.g., Rosenbusch et al., 2020) and even to detect construct identities and ameliorate the jingle/jangle problem in theory building (e.g., Larsen and Bong, 2016).

While the salient points of several of the articles presented in this Research Topic were semantically similar to prior literature, several others were more diverse (see Figure 1).

**Figure 1:**
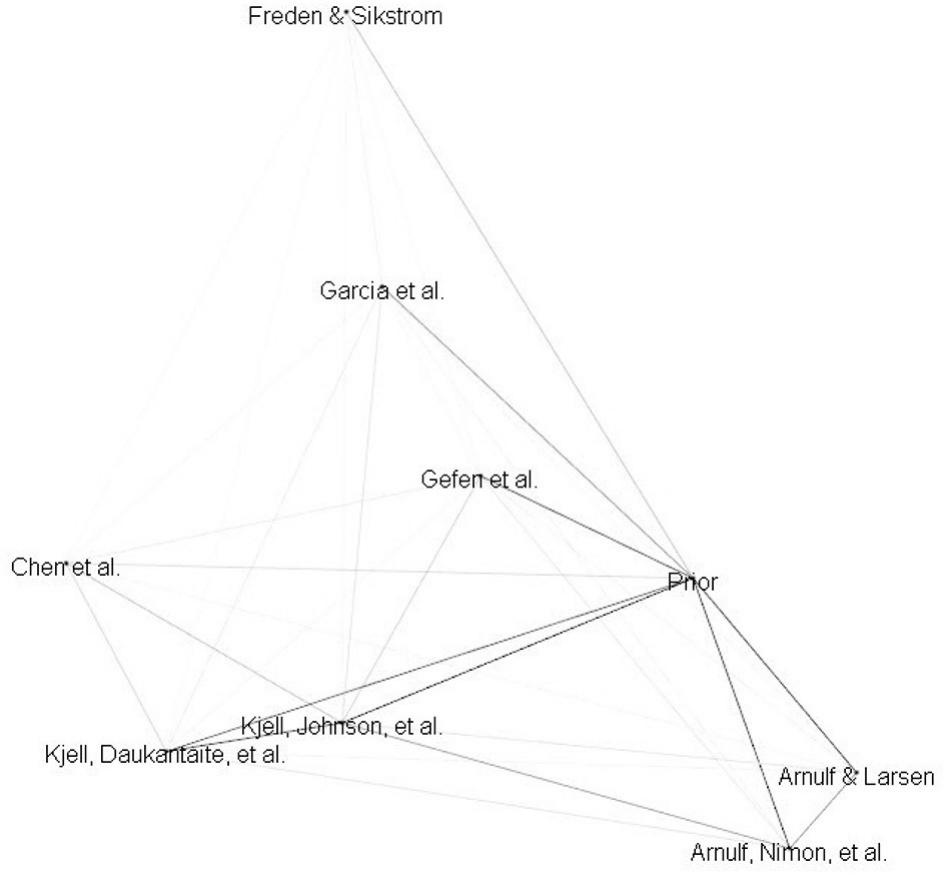
3D-Plot of Research Topic and prior literature abstracts semantic similarity. Prior encompasses the literature reviewed in the editorial not including the articles contributing to the Research Topic. Darkness of lines represents the magnitude of the cosines resulting from conducting LSA on the abstracts in the Research Topic and prior literature.

Arnulf and Larsen and Arnulf et al. are arguably most similar to the body of literature previously reviewed. In both articles, LSA of survey items predicted survey responses to varying degrees. Arnulf and Larsen's research questioned the capability of traditional survey responses to detect cultural differences. Observed differences in the semantically driven patterns of survey responses from eleven different ethnic samples appeared to be caused by different translations and understanding rather than cultural dependencies. Arnulf et al. similarly found that different score levels in prevalent motivation measures among 18 job types could be explained by differences in semantic patterns between the job types.

Gefen et al. conducted LSA on items sets associated with trust and distrust and found that the resulting distance matrix of the items yielded a covariance-based structural equation model that was consistent with theory.

Kjell O. et al. found that open-ended, computational language assessments of well-being were distinctly related to a theoretically relevant behavioral outcome, whereas data from standard, close-ended numerical rating scales were not. In a similar manner, Kjell K. et al. found that freely generated word responses analyzed with artificial intelligence significantly correlated with individual items connected to the DSM 5 diagnostic criteria of depression and anxiety.

Chen et al. manually annotated Facebook posts to assess social media affect and found that extraverted participants tended to post positive content continuously, more agreeable participants tended to avoid posting negative content, and participants with stronger depression symptoms posted more non-original content.

Garcia et al. applied LSA to Reuter news and Facebook status updates. In the case of the Reuter corpus, the past was devaluated relative to both the present and the future and in the case of the Facebook corpus, the past and present were devaluated against the future. Based on those findings, the authors concluded that people strive to communicate the promotion of a bright future and the prevention of a dark future.

Fredén and Sikstrom applied LSA to voter descriptions of leaders and parties and found that descriptions of leaders predicted vote choice to a similar extent as descriptions of parties.

Nimon provided a dataset of documents from *Taking the Measure of Work* and demonstrated how it could be used to build a LSA space.

As the NLP field continues to develop and mature and the opportunity to automatically transform open-ended data to quantifiable measures, one wonders to what degree the use of rating scales will be warranted in the future. Taken together, the applications demonstrated here go a long way in making free responses accessible to statistical treatment. Similarly, the NLP approaches even seem to allow statistical help in theory building, as the constructs themselves and their relationships with measurement scales may be modeled independently of response data. We invite readers to consider how NLP can advance and/or potentially replace the use of rating scales in the assessment of personality and attitudes.

## Author Contributions

All authors listed have made a substantial, direct and intellectual contribution to the work, and approved it for publication.

## Conflict of Interest

The authors declare that the research was conducted in the absence of any commercial or financial relationships that could be construed as a potential conflict of interest.

## Publisher's Note

All claims expressed in this article are solely those of the authors and do not necessarily represent those of their affiliated organizations, or those of the publisher, the editors and the reviewers. Any product that may be evaluated in this article, or claim that may be made by its manufacturer, is not guaranteed or endorsed by the publisher.
